# Mild Oxidative Stress Induces Redistribution of BACE1 in Non-Apoptotic Conditions and Promotes the Amyloidogenic Processing of Alzheimer’s Disease Amyloid Precursor Protein

**DOI:** 10.1371/journal.pone.0061246

**Published:** 2013-04-17

**Authors:** Jiang-Li Tan, Qiao-Xin Li, Giuseppe D. Ciccotosto, Peter John Crouch, Janetta Gladys Culvenor, Anthony Robert White, Genevieve Evin

**Affiliations:** 1 Department of Pathology, The University of Melbourne, Parkville, Australia; 2 Neuropathology Laboratory, Mental Health Division, The Florey Institute of Neuroscience and Mental Health, The University of Melbourne, Parkville, Australia; 3 BIO21 Molecular Science and Biotechnology Institute, The University of Melbourne, Parkville, Australia; Massachusetts General Hospital, United States of America

## Abstract

BACE1 is responsible for β-secretase cleavage of the amyloid precursor protein (APP), which represents the first step in the production of amyloid β (Aβ) peptides. Previous reports, by us and others, have indicated that the levels of BACE1 protein and activity are increased in the brain cortex of patients with Alzheimer’s disease (AD). The association between oxidative stress (OS) and AD has prompted investigations that support the potentiation of BACE1 expression and enzymatic activity by OS. Here, we have established conditions to analyse the effects of mild, non-lethal OS on BACE1 in primary neuronal cultures, independently from apoptotic mechanisms that were shown to impair BACE1 turnover. Six-hour treatment of mouse primary cortical cells with 10–40 µM hydrogen peroxide did not significantly compromise cell viability but it did produce mild oxidative stress (mOS), as shown by the increased levels of reactive radical species and activation of p38 stress kinase. The endogenous levels of BACE1 mRNA and protein were not significantly altered in these conditions, whereas a toxic H_2_O_2_ concentration (100 µM) caused an increase in BACE1 protein levels. Notably, mOS conditions resulted in increased levels of the BACE1 C-terminal cleavage product of APP, β-CTF. Subcellular fractionation techniques showed that mOS caused a major rearrangement of BACE1 localization from light to denser fractions, resulting in an increased distribution of BACE1 in fractions containing APP and markers for trans-Golgi network and early endosomes. Collectively, these data demonstrate that mOS does not modify BACE1 expression but alters BACE1 subcellular compartmentalization to favour the amyloidogenic processing of APP, and thus offer new insight in the early molecular events of AD pathogenesis.

## Introduction

BACE1 plays a critical role in the pathogenesis of Alzheimer’s disease. By mediating β-secretase cleavage of the amyloid precursor protein (APP) it initiates production of amyloid β (Aβ) peptides [1]. BACE1 cleavage of APP generates the soluble APP N-terminal fragment, sAPPβ, and a membrane-tethered C-terminal fragment of 99 amino acids (β-CTF or C99), which undergoes further processing by γ-secretase to release the APP intracellular domain (AICD) and Aβ fragments. BACE1 cleavage represents the rate-limiting step in Aβ formation. Accumulation of Aβ, which may result from its overproduction or defective clearance, causes formation of toxic fibrils and aggregated species responsible for neurodegeneration [2], [3]. Therefore, BACE1 represents a rational therapeutic target for AD treatment. Furthermore, *post-mortem* analysis of brain samples from AD patients has shown increased levels of BACE1 protein and enzymatic activity in cortical regions, but usually no change in mRNA levels [4], [5], [6], [7]. Thus, elucidating the mechanisms that regulate BACE1 cellular levels is fundamental for a better understanding of AD pathogenesis, in particular of the initial events that trigger Aβ production.

A variety of stress factors can induce BACE1 expression in cellular and animal models. These include oxidative stress [8], energy deprivation [9], as well as hypoxia and ischemic injury [10], [11], [12], [13], [14], and inflammation [15], [16], [17], which have been recently reviewed in the context of AD pathology [18]. Oxidative stress (OS) is particularly relevant to AD [19] and is a salient feature of neurodegeneration linked to the ageing process that is reflected by formation of protein carbonyl derivatives, lipid peroxidation and DNA damage [20]. OS-related protein and lipid modifications have been observed in various regions of the AD brain [21], [22]. Also, lipid peroxidation has been correlated with an increase in BACE1 activity in the sporadic AD brain [23]. Mounting evidence supports that OS accompanies AD pathogenesis [24], [25] and that it may represent the earliest event of the disease process [26], as it precedes biochemical changes characteristic of AD, such as tangle formation [27].

Previous experimental studies have investigated the effect of oxidative agents on BACE1 expression. Using a cellular luciferase reporter system, Tong et al demonstrated that BACE1 gene expression could be induced by hydrogen peroxide [28]. This was corroborated by studies showing that treatment of differentiated neuroblastoma cell lines with H_2_O_2_ and 4-hydroxynonenal increased BACE1 protein and mRNA levels [29], [30], [31], [32]. The induction of BACE1 expression by OS has also been demonstrated in rodent primary cortical cultures, using H_2_O_2_ [33], [34] and 4-hydroxynonenal [35]. OS-induced BACE1 expression can be mediated by the stress-activated protein kinase pathway, through activation of c-Jun N-terminal kinases (JNK) and p38 mitogen-activated protein kinase [29], [32], [36] as well as by activation of the eukaryotic translation initiation factor-2α (eIF2α) [37]. The finding that activation of eIF2α is increased in the frontal cortex of AD patients, and correlates with elevated BACE1 protein levels further supports the latest mechanism [37]. The PKR-eIF2α pathway is involved in apoptotic pathways [37], [38], [39], [40]. Furthermore, the induction of apoptosis also favours BACE1 accumulation in the endosome, as the adaptor protein, GGA3, which controls BACE1 trafficking to the lysosome for degradation, is sensitive to caspase-3 cleavage [41],[42],[43]. Caspase-3 activation has been reported to accompany increased BACE1 expression in cells treated with 4-hydroxynonenal [29]. Thus, the effect of OS on BACE1 expression has been largely associated with cell death events.

In the present study, we have examined alterations in endogenous BACE1 expression in response to mild levels of OS by subjecting mouse primary cortical neurons to non-toxic levels of oxidants. We have established mild oxidative stress (mOS) conditions, wherein oxidant treatment significantly increased levels of intracellular free radicals and activation of stress kinase without compromising cell viability. We showed that mOS treatment did not alter BACE1 levels. Notably, we found that mOS triggered a change in BACE1 compartmentalization that increases its colocalisation. with APP and its amyloidogenic processing.

## Materials and Methods

### Materials

All tissue culture reagents were purchased from Life Technologies, Mulgrave VIC, Australia. Chemicals were of tissue culture grade and were obtained from Sigma-Aldrich, Sydney, Australia, unless otherwise stated.

### Mouse Primary Cell Culture

Briefly, cortices from C57BL/6 embryonic mice at day 14 were removed, dissected free of meninges, dissociated in 0.025% (w/v) trypsin in Krebs Buffer (0.126 M NaCl, 2.5 mM KCl, 25 mM NaHCO_3_. 1.2 mM NaH_2_PO_4_. 1.2 mM MgCl_2_. 2.5 mM CaCl_2_, pH 7.2) and incubated in a shaking water-bath for 20 min at 37°C to dissociate the connective tissue. Then, 0.008% w/v DNase I (Roche Applied Science, Castle Hill, NSW, Australia)) and 0.026% w/v soybean trypsin inhibitor (Sigma, St. Louis, Missouri, USA) in 10 mL Krebs solution were added to neutralize trypsin. Fragmentation of the tissue precipitate was hastened by gentle inversion followed by centrifugation at 259×g (Beckman Coulter, Allegra 21R) for 3 min. The supernatant was aspirated and the cell pellet resuspended in 1 mL of DNase I/SBTI supplemented Krebs solution by passing thirty times through a blunt filtered tip. The resulting single cell suspension was diluted out in Krebs buffer solution and centrifuged to sediment the cells. The supernatant was aspirated and the cells resuspended in plating medium (Minimum Essential Medium, supplemented with 2 mM L-Glutamine, 0.22% v/v NaHCO_3_, 0.01 mg/mL Gentamicin, 10% v/v foetal calf serum and 5% v/v horse serum) and counted using a haemocytometer. The primary cortical cells were seeded onto poly-D-Lysine coated tissue culture dishes and cultured for up to 4 hours. Then, the plating medium was aspirated and the cells maintained in Neurobasal medium supplemented with 0.2 mM L-Glutamine, 0.01 mg/mL Gentamicin and B27 Supplement.

### Induction of Oxidative Stress in Mouse Primary Cortical Cell cultures

Mouse primary cortical cells were plated in 12-well poly-D-Lysine-coated tissue culture plates at a density of 8×10^5^ cells per mL, and cultured for 6 days before treatment with H_2_O_2_ (Analytical Reagents) at a final concentration of 10, 20, or 40 µM for 6–7 h. Working H_2_O_2_ concentrations were prepared in culture medium supplemented with B27 Minus Antioxidants in dark 15 mL tubes to minimise oxidant degradation by light. Control untreated cells were cultured in parallel.

### Assessment of Cell Viability by the MTT Assay

The 3-(4,5-dimethylthiazol-2-yl)-2,5-diphenyltetrazolium bromide (MTT) solution was prepared in sterile PBS at a concentration of 5 mg/mL and diluted 100 times in the culture medium. MTT was added into conditioned medium 30 min before the end of oxidative stress treatments; the cells were returned to the incubator until formazan purple coloration became visible. The conditioned medium was aspirated and the cells were lysed in DMSO for measuring absorbance at 540 nm using a Wallac VICTOR™ 3 Multilabel Counter (Perkin Elmer, Glen Waverley VIC, Australia). Cell viability was calculated as the ratio of absorption value for treated versus control cells and expressed as a percentage.

### Assessment of Cellular Oxidative Stress by DCF Assay

After treatment, cells were washed with PBS and incubated in culture medium supplemented with 50 µM 2′,7′-dichlorofluorescin-diacetate (DCFH-DA) for 40 min at 37°C. Next, the cells were washed with PBS and lysed in 0.1 M Tris-HCl pH 7.5 supplemented with protease inhibitor cocktail (P8340; Sigma) and 1% Triton X-100. Dichlorofluorescein (DCF) fluorescence was measured at 485/535 nm in a Wallac VICTOR™ 3 Multilabel Counter. The fluorescence values were normalised to the protein concentration in cell lysates, as determined by the Bicinchoninic assay (BCA; Pierce, Thermo Fisher Scientific Australia), and the data expressed relative to untreated cells.

### Cell Lysate Preparation

Cells were lysed using RIPA buffer (50 mM Tris-HCl pH 7.4, 150 mM NaCl, 1% NP-40, 0.5% sodium deoxycholate, 0.1% SDS, supplemented with PhosSTOP phosphatase inhibitor cocktail (Roche), P8340 protease inhibitor cocktail, and 0.05 mg/mL DNase I (Roche)). The lysate was centrifuged at 14,000×g for 5 min at 4°C and protein concentration was determined by BCA.

### Sodium Dodecyl Sulfate-Polyacrylamide Gel Electrophoresis and Immunoblotting

Samples (20 µg protein) were denatured in Laemmli sample buffer (62.5 mM Tris-HCl pH 6.8, 25% glycerol, 2% SDS, 0.01% bromophenol blue, 5% 2-mercaptoethanol). For BACE1 detection, samples were heated at 55°C for 10 min, whereas for analysis of other proteins, samples were heated at 95°C for 5 min. Proteins were separated 8.5% Tris-Glycine gels using a Mini Protean III system (Bio-Rad, Gladesville NSW, Australia) and electrophoresed at 40 mA/gel in 25 mM Tris, 192 mM Glycine, 0.1% SDS buffer, followed by transfer to nitrocellulose membrane (Bio-Rad) for 60 min at 600 mA in 25 mM Tris, 200 mM Glycine buffer containing 20% methanol. The 10–20% Novex Tris-Tricine gels (Life Technologies) were chosen to separate proteins of molecular weight lower than 40 kDa and were used following the manufacturer’s protocol. For APP-CTF detection, blots were boiled in PBS for 5 min before blocking. Western blot antibodies were as follows: p38 MAP kinase and phospho-specific p38 MAP kinase antibodies, BACE1 rabbit monoclonal D10E5, full-length (pro-) caspase-3, (from Cell Signaling Technology, Danvers, MA, USA)), transferrin receptor (Life Technologies), GGA3, TGN38 and EEA1 (Santa Cruz Biotechnology, Santa-Cruz, CA, USA). APP mouse monoclonal 22C11 and C-terminal rabbit polyclonal 369 were gifts from Prof K. Beyreuther and Prof S. Gandy, respectively. The blots were developed using West Dura chemiluminescence reagent (Pierce, Thermo Scientific, Australia) and signals captured with a LAS-3000 Imager (FUJIFILM). For reprobing membranes, antibodies were stripped by incubating the blots at 55°C for 15 min in 100 mM 2-mercaptoethanol (Bio-Rad), 2% SDS, 63.5 mM Tris-HCl pH 6.7, before repeating the immunoblotting procedure with another antibody.

### Subcellular Fractionation

Mouse primary cortical cells were grown on 100 mm diameter poly-D-Lysine-coated dishes at a density of 10×10^6^ cells per dish and cultured for six days (6 DIV) before treatment. Three dishes were subjected to H_2_O_2_ treatment, and three other dishes were left untreated for use as controls. The cells from three identical dishes were pooled, resuspended in homogenisation buffer (0.32 M sucrose, 1 mM MgCl_2_, 10 mM Tris-HCl, pH 7.4) and lysed with a Dounce homogeniser, followed with 5 passages through a 25-gauge needle. The homogenate was centrifuged at 1,200×g for 15 min, at 4°C. The supernatant was adjusted to 1.4 M sucrose. Sucrose gradients were constructed in 17 mL ultracentrifuge tubes by pipetting successively, 2 mL of 2 M sucrose, 2 mL of the 1.4 M sucrose mixture, 3.4 mL of 1.2 M sucrose, and 9.5 mL of 0.8 M sucrose. The tubes were centrifuged for 16 h, at 100,000×g, at 4°C, in a SW32.1 rotor in an L8-80M ultracentrifuge (Beckman Coulter). Fractions were collected and stored at −80°C until use. Fractions were concentrated by methanol precipitation [44] and the protein precipitates dissolved in Laemmli sample buffer before SDS-PAGE analysis.

### RNA Isolation and RT-qPCR

Cells were lysed using TRIzol reagent (Life Technologies) and RNA prepared according to the manufacturer’s protocol. The RNA pellet was air-dried for 10 min, before dissolution in 12 µL of DNase- and RNase-free water (Life Technologies) with incubation for 10 min at 57°C. RNA concentration and purity was assessed by measuring the 260 nm to 280 nm absorbance ratio (A_260/280_) using a NanoDrop 3300 fluorospectrometer (Thermo Scientific). The ratio obtained for RNA samples was ∼2. The RNA samples were distributed into aliquots and stored at −80°C. RNA was reverse-transcribed using the High-Capacity cDNA Reverse Transcription kit (Applied Biosystems, Life Technologies), which primed first strand synthesis with random primers. The reaction consisted of 2 µL 10× Reverse Transcription buffer, 4 mM dNTP mix, random primers, 50 U MultiScribe™ Reverse Transcriptase in a total volume of 10 µL plus 2 µg RNA in 10 µL nuclease-free water (Ambion, Life Technologies). The reactions were placed in a Mastercycler thermal cycler (Eppendorf), programmed for cycles at 25°C for 10 min, 37°C for 120 min and 85°C for 5 min. TaqMan® Gene Expression Assays (Applied Biosystems) were used for detecting the mouse BACE1 transcript (Assay ID Mm00478664_m1), and the mRNA transcripts of the house keeping genes actin (Assay ID Mm00607939_s1) and β2-macroglobulin (Assay ID Mm00437762_m1). In each 20 µL TaqMan® reaction, a volume containing 1 ng of cDNA (A_260/280_ ∼1.8) was mixed with 1 µL 20X TaqMan® Gene Expression Assay, 10 µL 2X TaqMan® Gene Expression Master Mix and nuclease-free water. Control reactions substituting cDNA with nuclease-free water were set up concurrently. Quantitative real-time PCR was performed using the Rotor-Gene™ 6000 (Corbett Life Science) with the conditions as follows: 50°C for 2 min proceeded by 95°C for 10 min, then 40 cycles of 15 s at 95°C and 60 s at 60°C. Data were analysed with Rotor-Gene 6000 Series software version 1.7 to determine the threshold cycle (C_T_), which used no cDNA template control reactions to establish the background signal of the assays. The 2-^ΔΔ^CT comparative method was used to determine the fold differences of BACE1 gene expression between treated and untreated cells [45]. BACE1 gene expression was normalised to either actin or β2-macroglobulin.

### Immunofluorescence Microscopy

Mouse primary cortical cells were plated at a density of 8×10^4^ cells per well on poly-D-Lysine-coated glass cover slips placed in 24-well plates, and treated as above. Cells were permeabilised by incubation with 0.1% Triton X-100 in PBS for 10 min. Then, the coverslips were blocked overnight with 10% v/v normal goat serum (NGS; Invitrogen) in PBS, before successive 1 h incubation with each primary antibody and Alexa Fluor-labelled secondary antibodies (Invitrogen), diluted in blocking buffer. Nuclei staining was carried out by incubation with 300 nM DAPI (4′,6-diamidino-2-phenylindole) in PBS for 5 min. Cells were visualized using a Leica (model DM IRB) inverted microscope, and images captured with a Zeiss Axiocam HRc camera. Images were superimposed using the Axio Vision software (Carl Zeiss) for assessment of colocalisation.

### Statistical Analysis

All experiments were performed at least three times, either with duplicates or triplicates. Statistical analyses were carried out using the SPSS statistics software version 17 (IBM). Comparison of the means was performed by One-way ANOVA (significant when p<0.05), followed by *post-hoc* analysis using the Tukey’s HSD (honestly significant difference) test or, when the Levene’s test indicated unequal variances (p<0.05), with the Games-Howell test. Error bars on the graphs correspond to the standard deviation.

## Results

### Optimization of mOS Conditions in Mouse Primary Cortical Cultures

**Figure 1:**
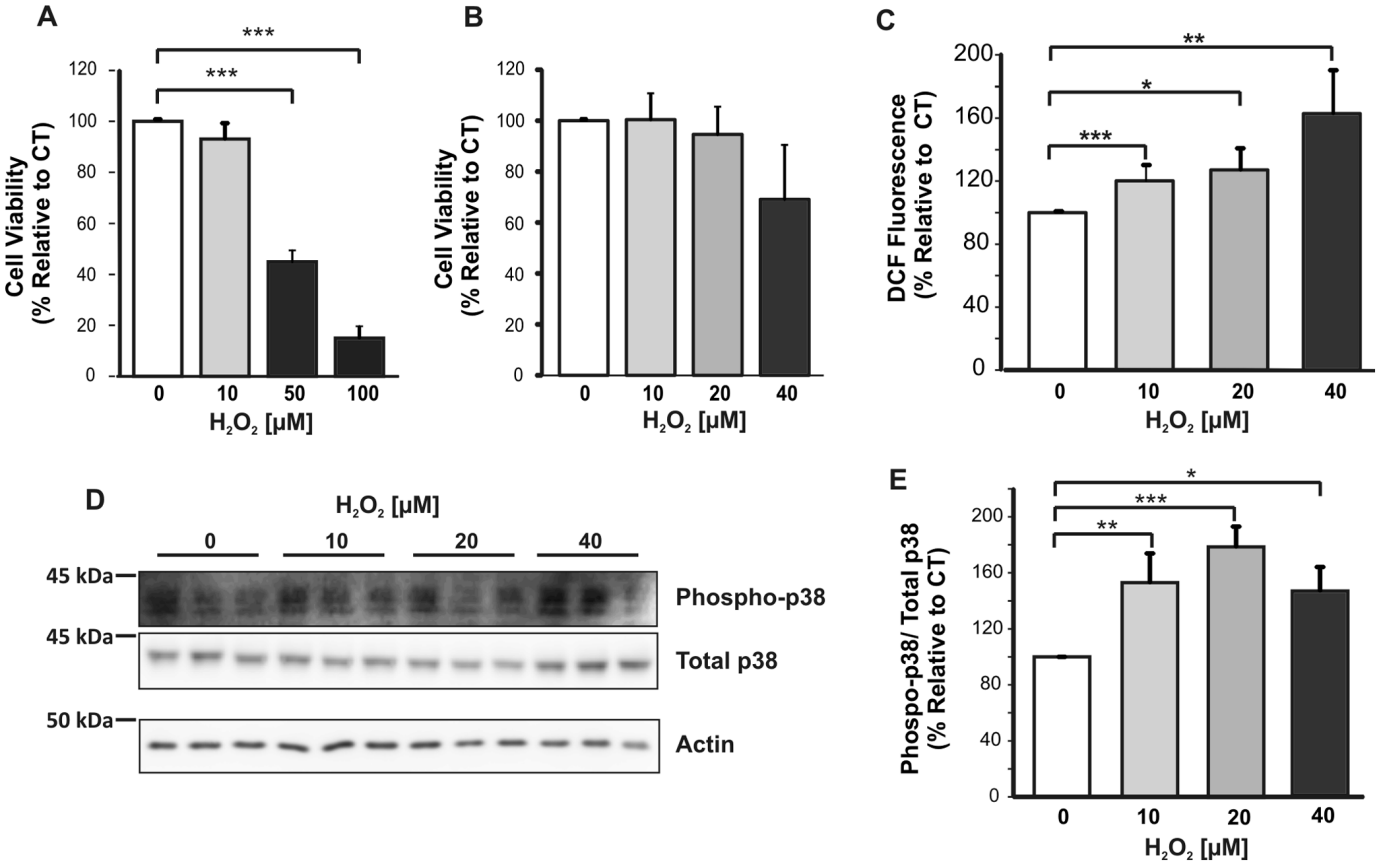
Establishment of mOS conditions for treatment of mouse primary cortical cultures. Cortical cells were treated for 6 h with various concentrations of H_2_O_2_. A. MTT assay of primary cortical cells treated with up to 100 µM H_2_O_2_ (n = 3) showed that viability decreased significantly by treatment with 50 and 100 µM H_2_O_2_. B. MTT assay of cells treated with 10–40 µM H_2_O_2_ (n = 6) showed no significant loss in viability compared to untreated cells. C. DCF assay of lysates of cells treated with 10–40 µM H_2_O_2_ and untreated controls. DCF fluorescence values were normalised for protein concentration in the lysates. Experiments were performed three times. D. Immunoblot of phosphorylated p38 and total p38 using 20 µg of cell lysates. E. Quantitative analysis of phospho-p38 and total p38 band densities from three separate blots. Data were analysed with ANOVA, and the Tukey’s HSD (A and E) and Games-Howell (B and C) *post-hoc* tests. *p<0.05; **p<0.001, ***p<0.005. Error bars represent the standard deviation.

First, we established conditions of mild oxidative stress that did not significantly compromise the cell viability. The MTT assay was performed to assess mitochondrial function of primary cortical cultures after a 6 h treatment with various concentrations of H_2_O_2_ (Figure 1A). A statistically significant reduction in cell viability was observed, with 55%±2% (*p<0.001*) increase in cell death after treatment with 50 µM H_2_O_2,_ and 85%±4% *(p<0.001*) increased cell death after treatment with 100 µM H_2_O_2_. Therefore, a lower range of H_2_O_2_ concentrations was trialled and treatment of primary cortical cultures with 10–40 µM H_2_O_2_ for 6 h did not cause any significant decrease in cell viability (Figure 1B).

Next, the levels of intracellular reactive oxygen species were determined using the 2′,7′-dichlororofluorescein (DCF) assay to confirm that the cells were undergoing oxidative stress [46]. A significant dose-dependent increase in intracellular free radicals was observed in primary cortical cells treated for 6 h with 10–40 µM H_2_O_2_ (Figure 1C), with a rise of 20%±6% after 10 µM H_2_O_2_ treatment *(p = 0.002)*, 27%±15% after treatment with 20 µM H_2_O_2_ *(p = 0.024)*, and 63%±26% after treatment with 40 µM H_2_O_2_ *(p = 0.007)*. As a significant accumulation of intracellular free radicals would only occur when endogenous antioxidants have been overwhelmed, the MTT and DCF data demonstrated that 10–40 µM concentrations of H_2_O_2_ could induce mOS in viable mouse primary cortical cells.

As a further indication that mouse primary cortical cells treated with 10–40 µM H_2_O_2_ were undergoing stress, p38 stress kinase activation was examined by immunoblotting with a specific phospho-p38 antibody. The p38 mitogen-activated protein (MAP) kinase belong to a family of serine/threonine protein kinases that play an important role in stress-activated pathways and become phosphorylated in response to stress stimuli [47]. Our data showed that lysates of primary cortical cells subjected for 6 h to low concentrations of H_2_O_2_ contained detectable levels of phosphorylated p38 kinase (Figure 1D). Statistical analysis of band signal density indicated a significant increase in p38 phosphorylation after treatment with 10 µM H_2_O_2_ (53%±20% increase; *p = 0.008),* 20 µM H_2_O_2_ (79%±14% increase; *p = 0.001)* and 40 µM H_2_O_2_ (46%±17% increase; *p = 0.031)* (Figure 1E). These data further support that our primary cortical cells experienced oxidative stress without significant loss in viability.

### BACE1 Expression Levels were not Altered by H_2_O_2_–induced mOS

**Figure 2:**
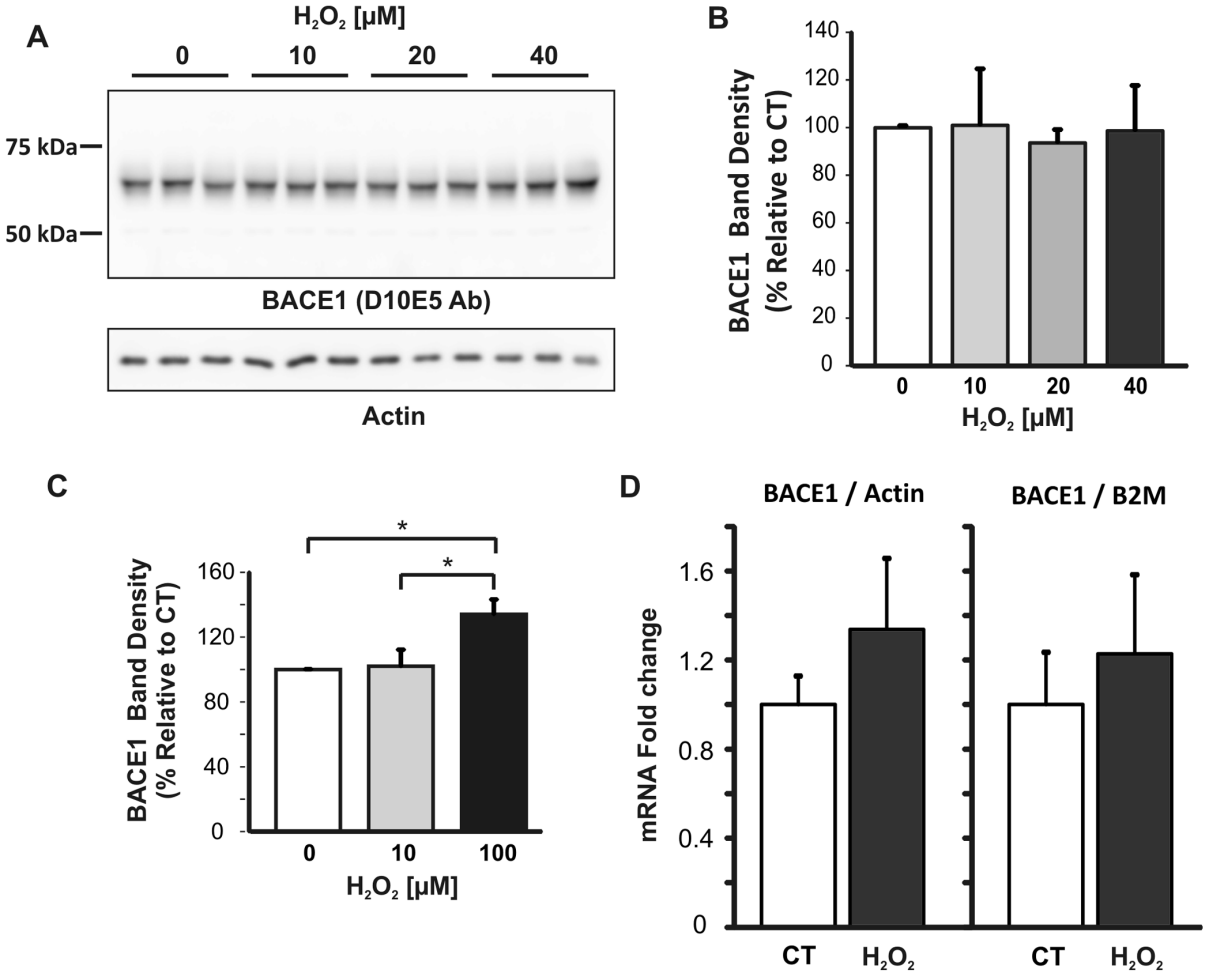
Analysis of BACE1 expression in primary neuronal cultures after mOS treatment. A. Representative BACE1 immunoblot. Cell lysates (15 µg/lane) were subjected to SDS-PAGE on 8.5% Tris-Glycine gels, followed by immunoblotting with D10E5 rabbit monoclonal antibody that targets BACE1 C-terminal region. Blots were re-probed for actin. B. Densitometry analysis of blots from three individual experiments. BACE1 signal density was normalised to actin. No statistical difference was found between treated and control cells. C. Densitometry analysis of BACE1 blots. BACE1 signal was significantly increased in cells treated with 100 µM H_2_O_2_ compared to untreated cells or cells treated with 10 µM H_2_O_2_. Data were analysed by One-way ANOVA, and the Games-Howell *post-hoc* test. Error bars represent standard deviation. D. RT-qPCR analysis of BACE1 mRNA. 2 µg of RNA was used for first strand synthesis and the resulting cDNA (1 ng) subjected to quantitative real-time PCR. Levels of BACE1 mRNA transcripts were calculated using the comparative C_T_ method relative to either actin or β2-macroglobulin (B2M) housekeeping genes. qRT-PCR assays were carried out in triplicate. Data represent average data of two independent treatments carried out in triplicate. Error bars represent range of the fold-differences, determined by incorporating the standard deviation of the ΔΔC_T_ value into the fold-difference calculation.

Next, BACE1 protein levels were analysed by immunoblotting with D10E5 BACE1 C-terminal antibody. BACE1 was detected as the expected 70 kDa signal (Figure 2A). Results from band density analysis showed that treatment of primary cortical cells with 10–40 µM H_2_O_2_ did not significantly alter BACE1 protein levels (Figure 2B). In contrast, treatment with 100 µM H_2_O_2_ (Figure 2C), a concentration which was previously shown to reduce cell viability by 85%±4% (Figure 1A), resulted in a 34%±9% increase in BACE1 protein signal *(p = 0.04).*

Quantitative real-time PCR (qPCR) was performed to assess possible changes in BACE1 mRNA levels in mouse primary cortical cells subjected to mOS. RNA was extracted with TRIzol, its purity checked, and it was used for reverse-transcription to generate cDNA for real-time PCR analysis using TaqMan gene expression assays. Actin and β2-macroglobulin (B2M) transcripts were chosen as the housekeeping gene controls. The mouse BACE1 TaqMan probe targets the junction between exons 2 and 3 of the BACE1 transcript, therefore signals generated by qPCR would not result from genomic DNA contamination. An increasing trend in BACE1 mRNA levels was observed in cells treated with H_2_O_2_, but this was not statistically significant when normalised to either actin or β2-macroglobulin mRNA (Figure 2D).

### Analysis of Caspase-3 Activation and GGA3 Levels

**Figure 3:**
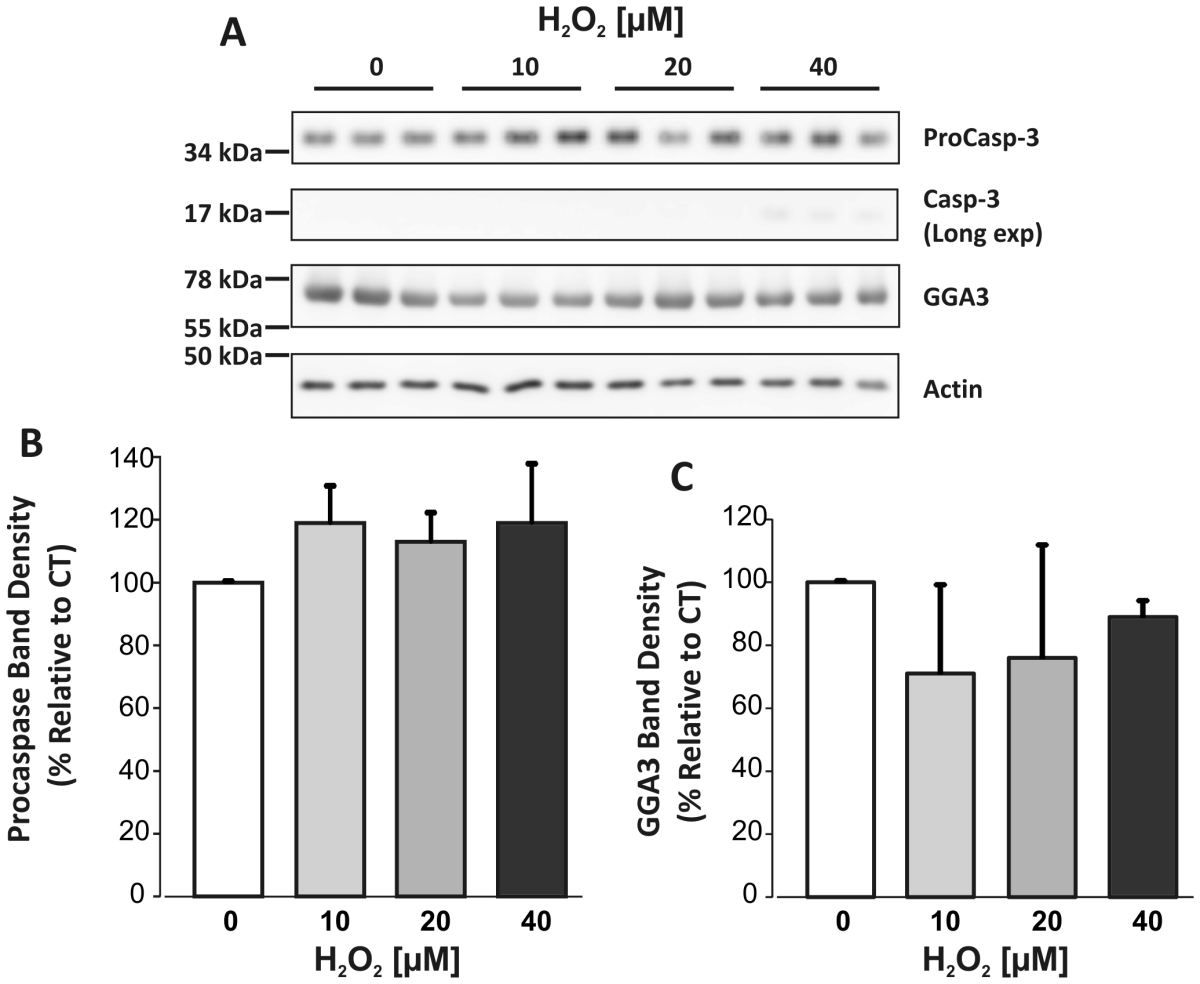
Immunoblot of caspase-3 and GGA3 in lysates of primary cortical cultures treated with H_2_O_2_. 20 µg of lysate was loaded per lane. A. Representative blots of caspase-3 and GGA3 from three experiments. Densitometry analyses of full-length proCaspase-3 35 kDa signal. C. Densitometry analysis of 75 kDa GGA3 signal. For both caspase-3 and GGA3, data were normalised to actin. One-way ANOVA indicated no significant change in levels of either protein in response to H_2_O_2_ treatments.

Cell lysates were analysed for caspase-3, which is activated by proteolytic cleavage under apoptotic conditions [48], and for GGA3, which controls BACE1 turnover. Indeed, it was shown that GGA3 is a substrate for capsase-3 processing and that its cleavage results in defective sorting of BACE1 for lysosomal degradation [41], [42]. Immunoblotting showed no change in the levels of either full-length procaspase-3 protein or its 17 kDa cleavage fragment that would indicate caspase-3 activation (Figures 3A–B). The caspase-3 cleavage fragment was detectable only after a long exposure time of the blot, albeit at barely visible levels after 40 µM H_2_O_2_ treatment, and was not consistently observed in all experiments. In agreement, no statistically significant change in GGA3 levels occurred in response to H_2_O_2_ treatment (Figure 3A and 3C). GGA3 cleavage products were not detectable (not shown). These data further support that the mouse primary neurons undergoing mOS were not undergoing apoptosis. However, when considering that caspase-3 is a downstream caspase that becomes activated after a cascade of proteolytic steps, we cannot definitely rule out that the apoptotic process had not been initiated in the cells. The steady levels of GGA3 protein were consistent with no alteration in BACE1 protein levels upon treatment of mouse primary neurons with non-toxic concentrations of H_2_O_2_.

### Analysis of APP C-terminal Fragments

**Figure 4:**
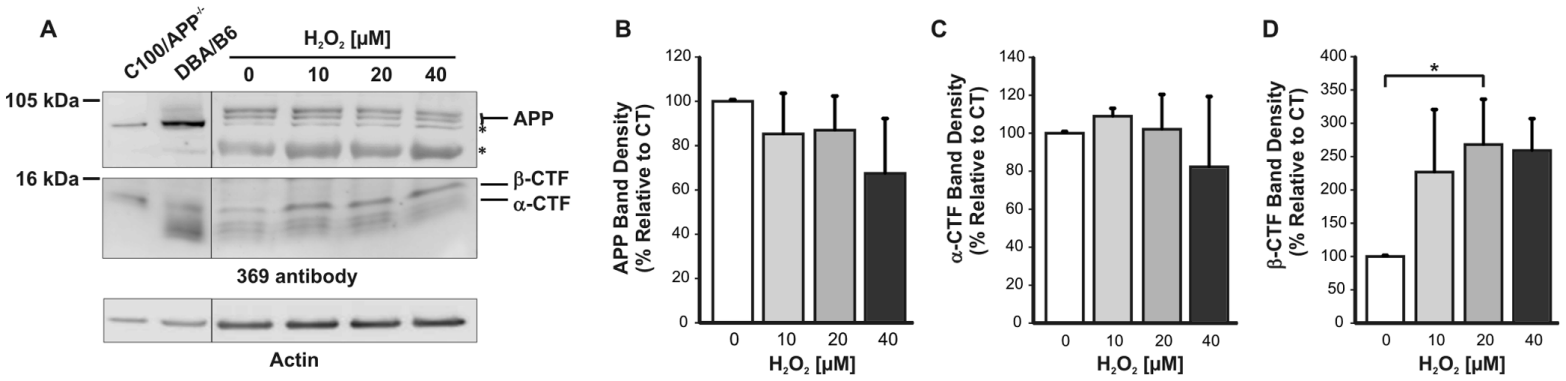
Quantitative analysis of APP C-terminal fragments in mouse primary cortical lysates after H_2_O_2_ treatment. A, 20 µg of lysate samples were electrophoresed on 10–12% Tris-Tricine gels and transferred to nitrocellulose. Blots were probed with anti-APP C-terminal antibody 369. Brain homogenates from an APP-null mouse expressing the last 100 C-terminal amino acids of human APP (C100/APP−/−) and from a DBA/B6 mouse (background strain to the transgenic) were co-electrophoresed as references for β-CTF and APP, respectively. The blots shown are representative of three separate experiments. The * denotes non-specific or uncharacterized bands. B. Densitometry analysis of APP signal relative to actin suggests a decreasing trend but this is not statistically significant. C. Densitometry analysis of α-CTF signal relative to actin also suggests a decreasing trend that is not statistically significant. D. Densitometry analysis of β-CTF signal relative to actin indicates a statistically significant increase after treatment with 20 µM H_2_O_2_ (p = 0.045). Experiments were performed three times with duplicates. Statistical significance was assessed using One-way ANOVA and the Tukey’s HSD *post-hoc* test used for pair-wise comparison.

We examined levels of the APP C-terminal product of BACE1 cleavage, β-CTF (C99) by immunoblotting with the polyclonal 369 antibody, which was raised against the whole cytosolic domain of APP [49]. Brain homogenate from a C100/APP^−/−^ mouse (an APP-null mouse line expressing the C-terminal one-hundred amino acids of human APP [50]), and that of a wild type mouse of the same DBA/B6 background were used as references for β-CTF and APP, respectively (Figure 4A). APP was detected as an ∼100 kDa doublet, which was absent in the C100/APP^−/−^ brain homogenate. C100 was detected as a prominent ∼12 kDa signal in the C100/APP^−/−^ homogenate. Other bands (marked by asterisks) could be due to non-specific signals or to the APP-like proteins and fragments, as the cytosolic domain is conserved among the APP family of proteins. A trend for decreases in full length APP (Figure 4B) and α-CTF (Figure 4C) was observed with increasing H_2_O_2_ concentrations. Conversely, the β-CTF signal was found to rise with H_2_O_2_ treatment (Figure 4D), with a significant 2.5-fold increase after 6 h treatment with 20 µM H_2_O_2_ *(p = 0.045).* Attempts to analyse changes in Aβ secretion in the cell conditioned media were unsuccessful, as the levels produced by mouse primary neurons are very low and were below the detection threshold of our sandwich ELISA that is less effective at measuring mouse Aβ than its human counterpart.

### Investigating Changes in BACE1 Subcellular Distribution in Response to mOS

Since mOS caused no change in either BACE1 mRNA or protein levels, but increased β-CTF, we reasoned that an alteration in the subcellular distribution of BACE1 might be promoting APP processing. Indeed, it was previously reported that oxidative stress treatment of NT2 neurons increased BACE1 localisation in the Golgi apparatus [31]. Thus, we aimed to investigate changes in subcellular distribution in response to mOS.

**Figure 5:**
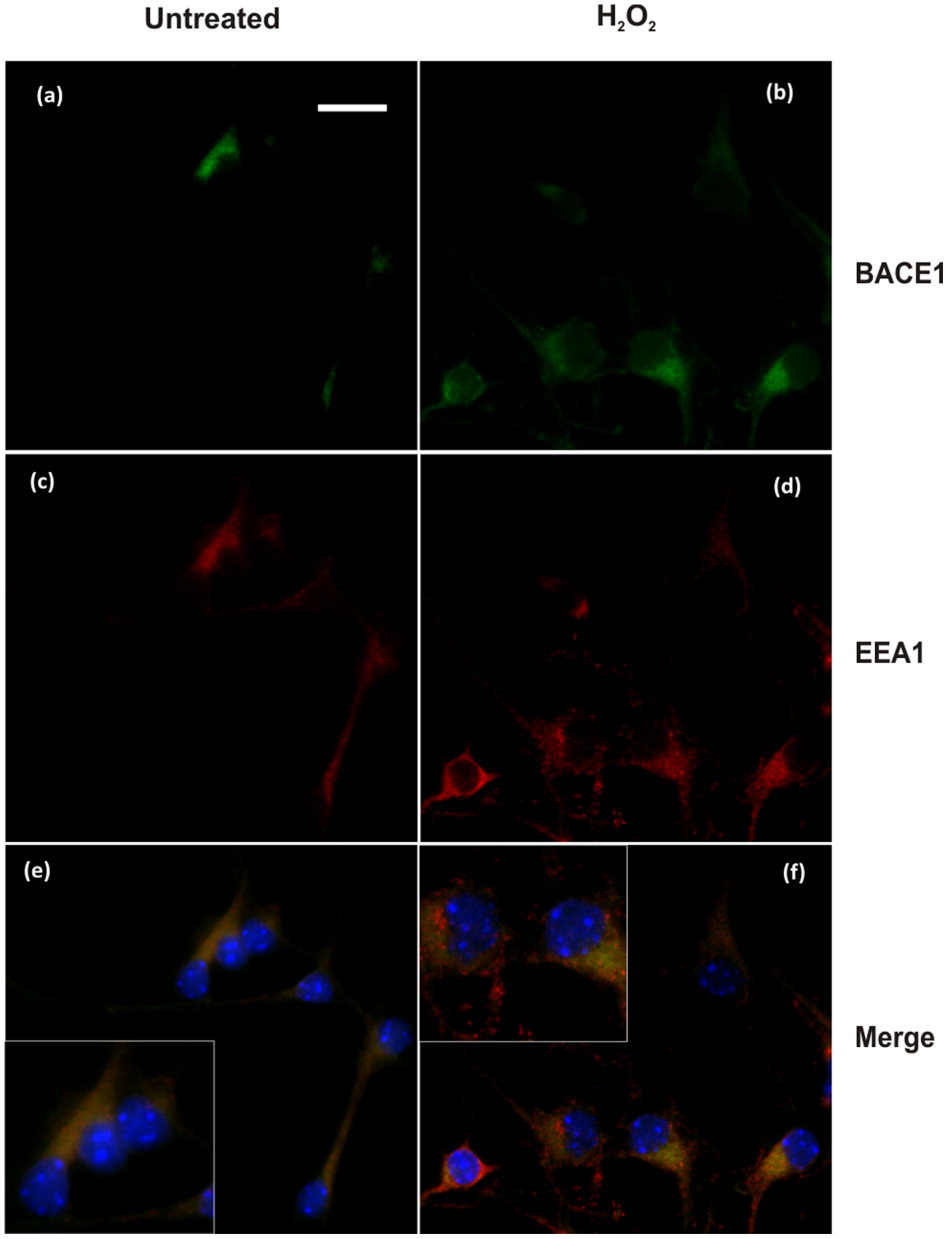
Immunofluorescence analysis of BACE1and colocalisation with early endosomal marker in mouse primary cortical neurons treated or untreated with H_2_O_2_. Immunolabelling of untreated cells (a,c,e) and H_2_O_2_-treated cells (b,d,f). Cells were stained for BACE1 (a,b) with mouse monoclonal antibody 61-3E7 and for early endosomal marker, EEA1 (c,d). Partial colocalisation of BACE1 and EEA1 was observed in both untreated cells (e), and treated cells (f). Bar = 20 µm.

**Figure 6:**
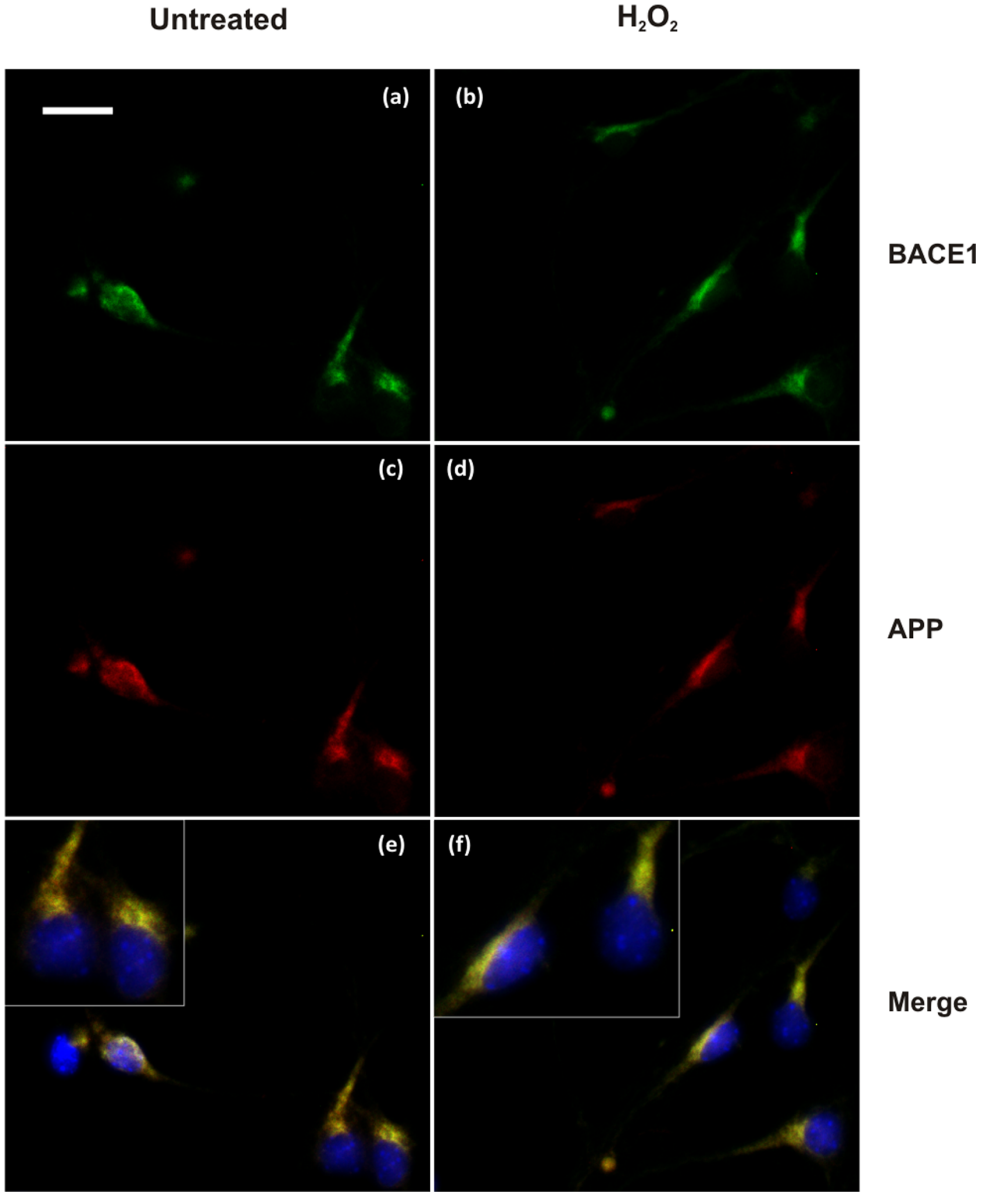
Immunofluorescence analysis of BACE1 and APP colocalisation. in mouse primary cortical neurons treated or untreated with H_2_O_2_. Immunolabelling of untreated cells (a,c,e) and H_2_O_2_-treated cells (B). BACE1 was labelled with mouse antibody 61-3E7 (a,b) and APP was revealed with rabbit antibody 369 (c,d). Since Ab 369 targets the cytosolic domain of APP, it can stain both APP full-length and C-terminal fragments. Colocalisation of BACE1 and APP was observed in untreated cells (e) and this colocalisation may be increased in the treated cells (f). Bar = 20 µm.

First, BACE1 cellular localisation was analysed by immunofluorescence. Labelling of endogenous BACE1 in primary cortical neurons using the C-terminal mouse monoclonal 61-3E7 antibody showed a perinuclear punctate staining and large colocalisation with the early endosome marker, EEA1 (Figure 5a and e). There was also a large degree of overlap with APP, as detected by C-terminal 369 antibody (Figure 6a and e). Treatment with 10 µM H_2_O_2_ did not markedly change BACE1 subcellular localisation, as overlap with the early endosomal marker was preserved (Figure 5b and f). Co-localisation with APP was possibly more extensive (Figure 6 b and f).

**Figure 7:**
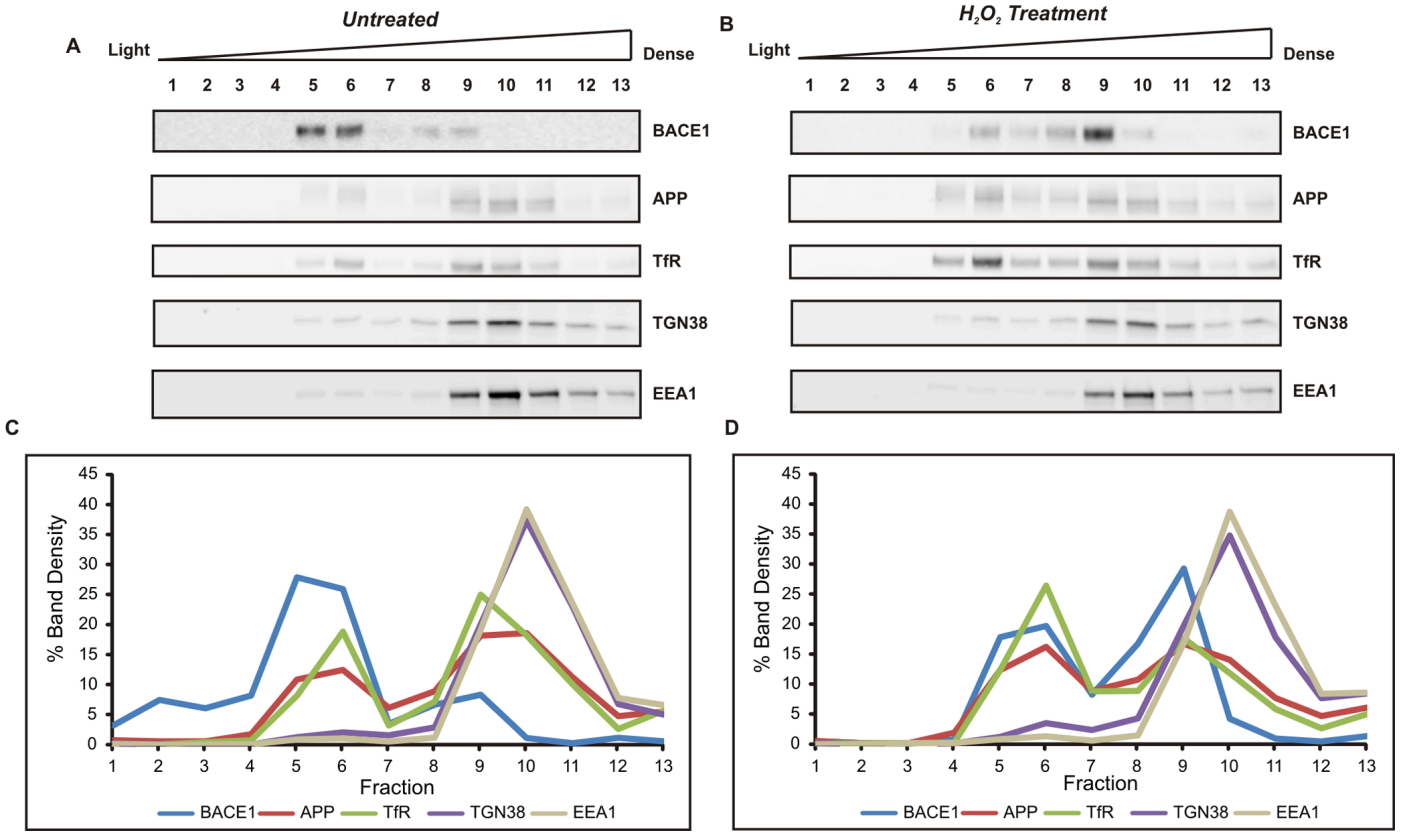
Subcellular fractionation of mouse primary cortical cells on a discontinuous sucrose density gradient. Cells were incubated for 6 h in the presence or absence of 40 µM H_2_O_2_ and lysed under isotonic conditions. Post-nuclear supernatants were centrifuged at 100,000×*g* on a discontinuous sucrose gradient. Fractions were analysed by immunoblotting with antibodies for BACE1 (D10E5), APP (22C11) and for organelle markers (Transferrin receptor, TfR for plasma membrane and endocytic vesicles; TGN38, for trans-Golgi; EEA1 for early endosomes). Signal density was determined for all fractions, and specific protein levels in each fraction were expressed as a percentage of the total signals from all thirteen fractions. Representative blots from two experiments with untreated cells (A) and H_2_O_2_-treated cells (B). Graphs (C and D) represent signal intensity % of each protein plotted against fraction number. Noticeable changes in BACE1 distribution were observed after H_2_O_2_-treatment, with BACE1 protein redistributing from light to denser fractions, enriched in EEA1 and TGN38 markers.

Next, we fractionated cellular organelles by differential centrifugation on a discontinuous sucrose density gradient. Fractions were analysed by western blotting for BACE1, APP, the transferrin receptor, which was used as a marker for plasma membrane and endocytic vesicles, and for the two organelle markers, EEA1 and TGN38. Representative blots are displayed in Figure 7. For each fraction, the band densitometry was expressed as the percentage of the sum of densities for all fractions. The graphs represent the average results of two separate experiments. In the cells that were not exposed to H_2_O_2_ (Figure 7A and 7C), over 70% of BACE1 was recovered in fractions of low density (5 and 6) and ∼20% in denser fractions (8 and 9). In contrast, APP was mostly distributed in dense fractions (∼60% in fractions 9–11), where the TGN38 and EEA1 markers were enriched, and only 17% of APP was recovered in the light fractions (5 and 6), together with BACE1. The distribution profile of the transferrin receptor closely resembled that of APP, as would be expected since both proteins follow similar trafficking routes, and both are present at the plasma membrane and recycling endosomes. After treatment with 40 µM H_2_O_2_, there was a marked change in the distribution of BACE1, as 49% of immunoreactivity was detected in a fraction of high density (number 9), which contained transferrin receptor, EEA1, and TGN38 (Figure 7B and 7D). A greater overlap with APP was also observed, with 10% shifting to light fractions 5–6. These data suggest that H_2_O_2_ treatment had caused the BACE1 protein to redistribute from low-density membrane vesicles, such as lipid rafts and perhaps recycling endosomes, to the trans-Golgi network and early endosomal compartments where APP is enriched. This redistribution would favour β-CTF production and be consistent with the increase in β-CTF that was observed in the cell lysates.

## Discussion

BACE1 plays a critical role in AD pathogenesis as it initiates the amyloid cascade. We hypothesized that BACE1 activity may be potentiated by low levels of oxidative stress, as occurring in the ageing brain. Our results demonstrate for the first time that non-toxic levels of oxidative stress can alter BACE1 cellular compartmentalization and increase APP β-CTF levels. Previous studies have reported that BACE1 levels are increased in brain cortex regions affected by amyloid deposition [4], [5], [7], [51], [52]. Also, BACE1 activity was reported to be elevated in the CSF of patients with prodromal AD [53]. In addition, experimental evidence supports that BACE1 activity increases with age and in response to various stress injuries of the brain related to ageing [54], [55], [56].

Previous studies have shown that induction of oxidative stress increases BACE1 protein levels in permanent cell lines [29], [30], [31], [36], [37], [57] and in primary neurons [33], [34], [35], and that this event is usually associated with apoptosis and cell death signalling [29], [41], [58], [59]. Two studies involving treatment of rat primary neurons with low concentrations of oxidants showed an increase in BACE1 protein and mRNA transcript [34], [35],but the authors did not provide data on the cell viability, therefore the contribution of apoptosis and other cell death pathways to these changes in BACE1 expression cannot not be fully evaluated. From a report by Kao et al. [33], BACE1 protein was increased in primary neuronal cultures that exhibited a marked cell loss after H_2_O_2_ exposure. This was recapitulated in our cell cultures, wherein only a toxic concentration of H_2_O_2_, which caused an ∼85% loss of primary neuronal cells, was capable of increasing endogenous BACE1 protein levels (Figure 1A and 2C). In addition, studies with permanent cell lines that showed an increase in BACE1 expression with oxidative stress also presented evidence that cell death mechanisms were operating, such as increased activation of caspase-3 [29] or of the pro-apoptotic protein kinase R [37]. Another study has also reported an oxidative stress induction of BACE1 expression in the presence of significant cell death [36].

Consistent with the cell viability data (Figure 1B), we found no supporting evidence that the apoptotic pathway was prevalent in our mouse primary cortical cells exposed to low concentrations of H_2_O_2_, as cleaved caspase-3 was hardly detectable (Figure 3). Furthermore, the GGA3 protein adaptor that controls BACE1 trafficking was apparently unaffected. Previously, caspase-3 was shown to cleave GGA3 under apoptotic conditions [41], hence GGA3 degradation would consequently disrupt BACE1 cellular trafficking and turnover by the lysosome. Increased levels of activated caspase-3 were also shown to accompany the increase in BACE1 expression observed in differentiated human NT2 neuroblastoma cells undergoing 4-hydroxynonenal-mediated oxidative stress [29]. However, other studies have also reported accumulation of BACE1 protein in the absence of cell death. The treatment of differentiated NT2 cells with combinations of either ascorbic acid/FeSO_4_ or H_2_O_2_/FeSO_4_ increased BACE1 protein levels without significantly compromising cell viability [31]. Also hypoxia-induced oxidative stress was reported to elevate BACE1 proteins in viable differentiated SK-N-BE neuroblastoma cells [13]. Differences in cell types or in the methods for inducing oxidative stress may account for different results. Guglielmotto and colleagues [13] reported that hypoxia treatment caused a biphasic increase of BACE1; the early phase occurred in viable cells and was associated with increased reactive oxygen species and stress kinase activation, whereas the later phase was observed concomitantly with activation of the hypoxia inducible factor-1α in cultures that exhibited significant cell death.

We have also evaluated the effect of oxidative stress induced by the alternative reagents, paraquat and linsidomine (SIN-1) (Methods S1). These reagents are known to produce oxidative stress through alternative mechanisms. Paraquat is a mitochondrial toxin, which generates the superoxide anion radical, whereas SIN-1 produces the peroxinitrite ion radical, which both represent an alternative source of reactive oxygen species. Similarly to the experiments carried out with hydrogen peroxide, we established paraquat and SIN-1 concentrations that induced mild oxidative stress in the cells, as demonstrated by the preservation of cell viability (Figure S1) and production of free radicals (Figure S2). We also demonstrated p38 activation (Figure S3). In these conditions, neither of these compounds caused an increase in BACE1 protein expression in our neuronal cultures, (Figure S4) or a change in GGA3 levels (Figure S5). Therefore, we concluded that in the absence of cell death activation process, low levels of oxidative radicals might not significantly modify the levels of BACE1 expression in mouse primary cortical cultures.

Immunofluorescence localisation studies suggested a greater colocalisation of BACE1 with the early endosomal marker, EEA1 in the cells that underwent mild oxidative stress (Figures 5 and 6 and Figure S6). We also observed an increase in β-CTF levels in the primary cultures treated with H_2_O_2_, which suggested a cellular response to promote the amyloidogenic processing of APP. Subcellular fractionation demonstrated a shift in the distribution of BACE1 protein from fractions of light density towards fractions containing APP and markers of the trans-Golgi network (TGN38) and early endosome (EEA1) (Figure 7). A previous study with differentiated NT2 neurons showed oxidative stress to substantially increase BACE1 colocalisation. with Golgi marker 58K [31]. Our own data may suggest that mOS treatment of primary neurons would cause the redistribution of BACE1 to TGN and early endosomes, which are cellular compartments where BACE1 was shown to reside [60], [61], [62], [63] and which provide an acidic environment optimal for BACE1 activity [64], [65], [66], [67]. Increased occurence of BACE1 in these compartments and its enhanced colocalisation. with APP would facilitate production of β-CTF, which is consistent with what was observed in our cells.

The intricate and regulated intracellular trafficking of BACE1 has been mostly studied in overexpressing systems (reviewed in [68]), where a small proportion of BACE1 was localised to the plasma membrane [60], [69]. The enrichment of BACE1 in fractions of low-density in the untreated cells is unlikely to represent plasma membrane accumulation and would rather suggest an association with lipid rafts and membrane vesicles rich in cholesterol. Compelling evidence supports that BACE1 undergoes recycling between TGN, plasma membrane, and endosomes, under the control of GGA adaptors [61], [70], [71] and retromer-associated proteins [61], [72]. A recent study has demonstrated its primary sorting to a specialized population of early endosomes, where it is secluded from APP [72]. This is also consistent with previous studies showing that the segregation of BACE1 to lipid raft membrane domains controls its access to APP [73], [74], [75], [76], [77]. Treatment with a non-cytotoxic concentration of H_2_O_2_ seems to induce the redistribution of BACE1 from these light membrane vesicles or raft domains to facilitate its interaction with APP in endosomal and TGN membranes.

The molecular mechanism inducing the redistribution of BACE1 upon oxidative stress will require further investigation. This may involve phosphorylation of BACE1 at serine 498, which was shown to mediate efficient trafficking between early and late endosomal compartments, or retrieval from early endosomes to the trans-Golgi network [78], [79]. Furthermore, BACE1 phosphorylation enhances an interaction with the GGA1 protein [63], [71], which facilitates anterograde trafficking from TGN to endosomal compartments. Although casein kinase 1 is a candidate for BACE1 phosphorylation [69], [79], a possible involvement of MAPK would be worth investigating. Another potential mechanism could involve free radical activation of the c-Src tyrosine kinase [80], [81], which mediates BACE1 internalization [82]. Besides being modulated by phosphorylation and dephosphorylation mechanisms, Src can also be activated by cysteine oxidation [80]. A possible link between c-Src and oxidative stress for BACE1 accumulation in early endosomes would thus warrant further investigation in the context of mild oxidative stress. The possible contribution of ER stress signalling in our mild oxidative stress model would also be worth exploring. ER stress has been linked to AD pathogenesis as activation of the unfolded protein response (UPR) pathway is observed in the AD brain [83], and its interplay with mitochondrial dysfunction and oxidative stress is being revealed [84], [85]. Several studies support that ER stress can potentiate BACE1 expression. Experimental induction of ER stress caused by energy deprivation increases BACE1 expression and elevates Aβ production by a mechanism that involves eIF2α phosphorylation [86]. Also, ER stress activates the nuclear factor NF-κB, which binds to BACE1 promoter to modulate its transcriptional activity [15], [16]. Whether ER stress may alter BACE1 subcellular compartmentalization would deserve investigation as the induction of ER stress in neuronal cells was shown to alter the trafficking of APP [87] and thus may also affect that of BACE1.

**Figure 8:**
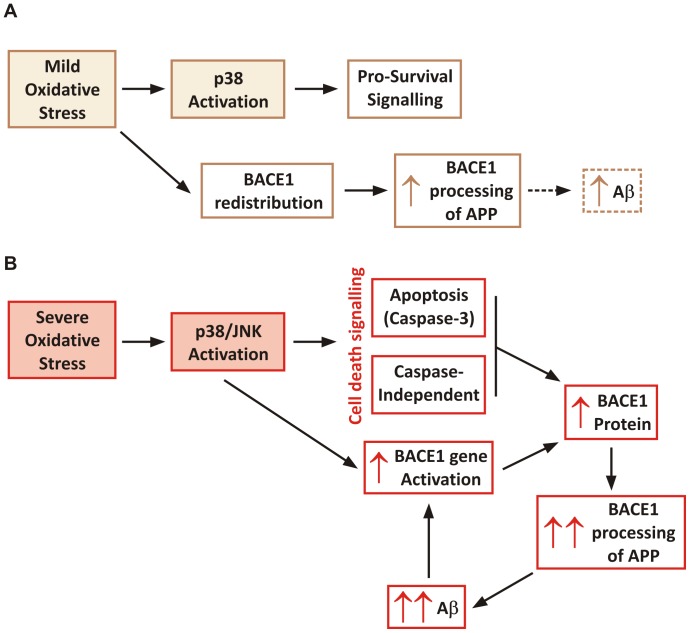
Schematic proposing a biphasic effect of oxidative stress on BACE1. A. Effect of mOS on BACE1 expression. mOS does not alter BACE1 levels but induces its subcellular redistribution to enhance colocalisation. with APP and favour β-CTF production. It may be speculated that this also leads to Aβ production. mOS also induces p38 stress kinase phosphorylation, which can trigger cell survival signaling. B. Effect of severe OS. This increases BACE mRNA and protein expression, thereby increasing APP amyloidogenic processing. MAP kinases also become activated, as well as apoptotic mechanisms. These events cause further cellular accumulation of BACE1, particularly in cellular compartments where APP is present, hence resulting in higher Aβ production and leading to formation of toxic species that induce further OS, by way of a feed-back mechanism.

To consider our results in the context of the literature that has reported increases in BACE1 mRNA and protein levels in response to oxidative stress, we would like to propose that oxidative stress may exert a dual effect on BACE1, depending on the stress intensity inflicted to neurons (Figure 8). In the first stage, low levels of oxidative stress would cause the redistribution of BACE1 and promote the amyloidogenic processing of APP. This would be accompanied by p38 activation, which under mild stress signals pro-survival mechanisms, and this would provide a recovery process for neurons undergoing mild oxidative injury. In the second stage, when oxidative stress is sustained or severe, apoptotic mechanisms would become activated and BACE1 expression would increase, thereby promoting a larger amount of Aβ to be produced. Aβ accumulation and aggregation would then cause further oxidative stress and exacerbate a process that would become overwhelming and ultimately result in neuronal death.

### Conclusion

By inducing oxidative stress with H_2_O_2_ in mouse primary cortical cultures we have observed that the levels of endogenous BACE1 would increase only in conditions associated with significant cell death. We showed that mild, non-lethal oxidative stress induced by low H_2_O_2_ concentrations caused changes in BACE1 cellular compartmentalization and increased colocalisation. with APP, and that this was accompanied by the increased production of the direct Aβ precursor fragment, β-CTF. Taken together, our data suggest that mild oxidative stress can promote the amyloidogenic processing of APP and trigger the amyloid cascade, and may therefore contribute to the early events of AD pathogenesis.

## Supporting Information

Figure S1 **Cell viability assay of mouse primary cortical cells treated with SIN-1 and paraquat.** Primary cortical cells were treated with SIN-1 for 24 h, or with paraquat fir 6 h, at the indicated concentrations. A. MTT assay shows no loss in viability of cells treated with SIN-1 at 2.5–10 µM concentrations. B. LDH assay of cells treated with paraquat. The increasing trend in LDH activity due to treatment with 100–400 µM paraquat concentrations was not statistically significant, suggesting no decrease in cell viability. Experiments were performed three times.
(TIF)

Figure S2 **DCF assay of cortical cells treated with SIN-1 and paraquat.** DCF fluorescence of cell lysate was normalised for protein concentration. Experiments were performed three times. The results show a significant increase in oxidative radicals in cells treated with 2.5, 5, and 10 µM SIN-1 (A) and in cells treated with 200 and 400 µM paraquat (B), indicating that the cells experienced oxidative stress.
(TIF)

Figure S3 **Immunoblot of phosphorylated p38 and total p38 in cortical cells treated with SIN-1 and paraquat.** Western blot was carried out with 20 µg of cell lysates. Blots for SIN-1 and paraquat treatments are displayed in (A) and (C), respectively. These are representative of three separate experiments. The results indicate that the ratio of phospho-p38/total p38 was elevated in cells treated with 5 and 10 µM SIN-1 (B), supporting activation of p38 stress kinase. Treatment with 400 µM paraquat also caused a significant increase in phospho-p38/total p38 ratio (D).
(TIF)

Figure S4 **Analysis of BACE1 expression in primary cortical cells treated with SIN-1 and paraquat.** Western blot was carried out with 20 µg of cell lysates using anti-BACE1 CT D10E5 antibody A. Representative immunoblot for SIN-1 treatment C. Representative immunoblot for paraquat treatment, respectively. Blots were re-probed for actin. B. and D. Densitometry analysis of blots for SIN-1 and paraquat treatments. The data represent averages of three individual experiments. BACE1 signal density was normalised to actin. No statistical difference was found between treated and control cells.
(TIF)

Figure S5 **Immunoblot of caspase-3 and GGA3 in cortical cells treated with SIN-1 and paraquat. 20 µg of lysate was loaded per lane.** A and D. Blots of caspase-3 and GGA3 for cells treated with SIN-1 and paraquat, respectively. B. Densitometry analysis of full-length pro-caspase-3 normalized to actin, for cells treated with SIN-1 (N = 3). C. Densitometry analysis of 75 kDa GGA3 signal normalized to actin, for cells treated with SIN-1 (N = 3). E. Densitometry analysis of full-length pro-caspase-3 normalized to actin, for cells treated with paraquat (N = 3). F. Densitometry analysis of 75 kDa GGA3 signal normalized to actin, for cells treated with paraquat (N = 3). There was significant change in pro-caspase 3 and GGA3 levels after either treatment with SIN-1 or paraquat at the indicated concentrations, suggesting that the cells were not undergoing apoptosis.
(TIF)

Figure S6 **Immunofluorescence analysis of mouse primary cortical neurons treated with SIN-1 and paraquat.** The left panel shows cells treated with SIN-1 and control. The right panel shows cells treated with paraquat and control. (a) and (b) show immunolabelling of BACE1 as punctate perinuclear staining (detected with mouse monoclonal antibody 61-3E7). (c) and (d) show detection of early endosomal marker EEA1. (e) and (f) are the merged images of BACE1 and EEA1. Partial colocalisation of BACE1 and EEA1 was observed in untreated cells (e), and this was increased in the cells treated with SIN-1 (left panel, f) or with paraquat (right panel, f). Bar = 20 µm.
(TIF)

Methods S1 (DOCX)
