# Correction: Mild Oxidative Stress Induces Redistribution of BACE1 in Non-Apoptotic Conditions and Promotes the Amyloidogenic Processing of Alzheimer’s Disease Amyloid Precursor Protein

**DOI:** 10.1371/annotation/da7ad86d-a5dc-4b03-981b-e44a2392c67b

**Published:** 2013-10-17

**Authors:** Jiang-Li Tan, Qiao-Xin Li, Giuseppe D. Ciccotosto, Peter John Crouch, Janetta Gladys Culvenor, Anthony Robert White, Genevieve Evin

Under sub-section "Mouse Primary Cell Culture", line 2,“0.025% w/v” should read as “0.016% w/v”

Under sub-section "Mouse Primary Cell Culture"The Krebs Buffer recipe indicated in parentheses should read as

(124 mM NaCl, 5.37 mM KCl, 1 mM NaH2PO4, 1 mM MgSO4, 14.4 mM D-Glucose, 24.9 mM HEPES and 0.0027 mM Phenol red, pH 7.4, supplemented with 0.3% w/v bovine serum albumin)

Under sub-section "Mouse Primary Cell Culture", lines 4 and 5 “0.008% w/v” should read as “0.08% w/v”, and “0.026% w/v” should read as “0.26% w/v”

Under sub-section "RNA Isolation and RT-qPCR", 2-ΔΔCT should read as 2-ΔΔ^CT^

