# Supplementary figures and images for: Mild Oxidative Stress Induces Redistribution of BACE1 in Non-Apoptotic Conditions and Promotes the Amyloidogenic Processing of Alzheimer’s Disease Amyloid Precursor Protein
Source: PLoS One. 2013 Apr 17;8(4):e61246. doi: 10.1371/journal.pone.0061246 (PMC3629182; doi:10.1371/journal.pone.0061246)

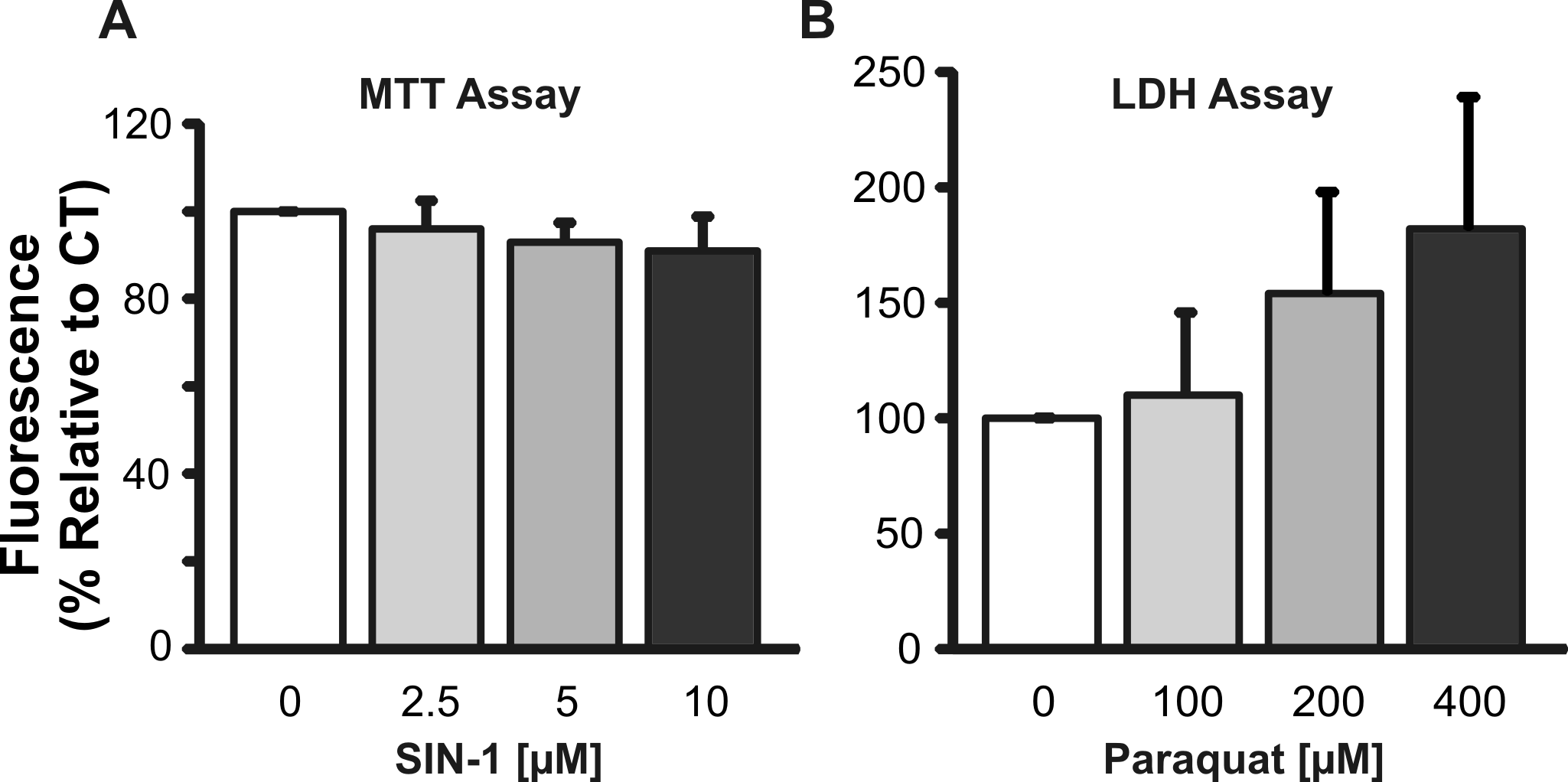

Supplement: Figure S1 — Cell viability assay of mouse primary cortical cells treated with SIN-1 and paraquat. Primary cortical cells were treated with SIN-1 for 24 h, or with paraquat fir 6 h, at the indicated concentrations. A. MTT assay shows no loss in viability of cells treated with SIN-1 at 2.5–10 µM concentrations. B. LDH assay of cells treated with paraquat. The increasing trend in LDH activity due to treatment with 100–400 µM paraquat concentrations was not statistically significant, suggesting no decrease in cell viability. Experiments were performed three times. (TIF) [file pone.0061246.s001.tif]

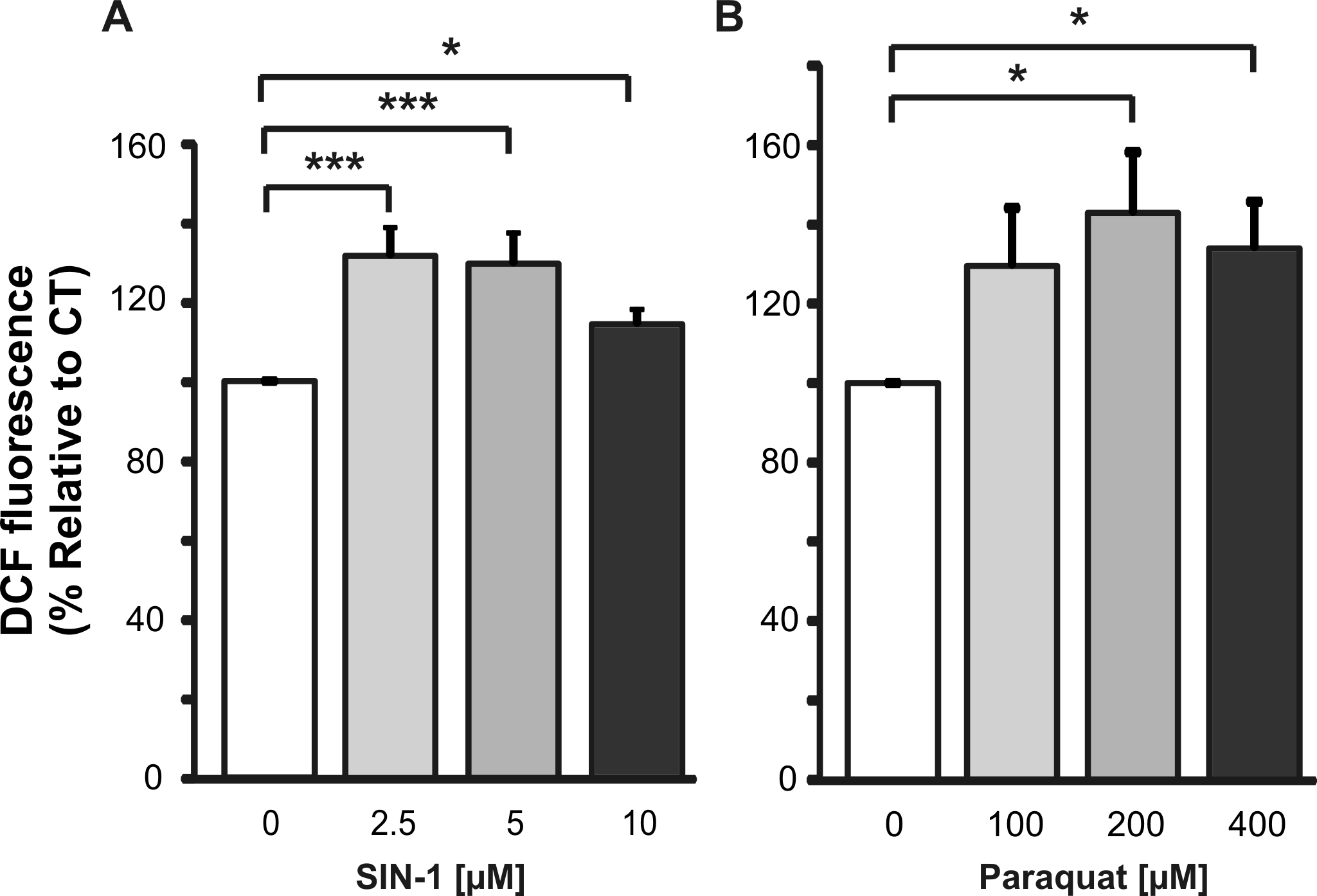

Supplement: Figure S2 — DCF assay of cortical cells treated with SIN-1 and paraquat. DCF fluorescence of cell lysate was normalised for protein concentration. Experiments were performed three times. The results show a significant increase in oxidative radicals in cells treated with 2.5, 5, and 10 µM SIN-1 (A) and in cells treated with 200 and 400 µM paraquat (B), indicating that the cells experienced oxidative stress. (TIF) [file pone.0061246.s002.tif]

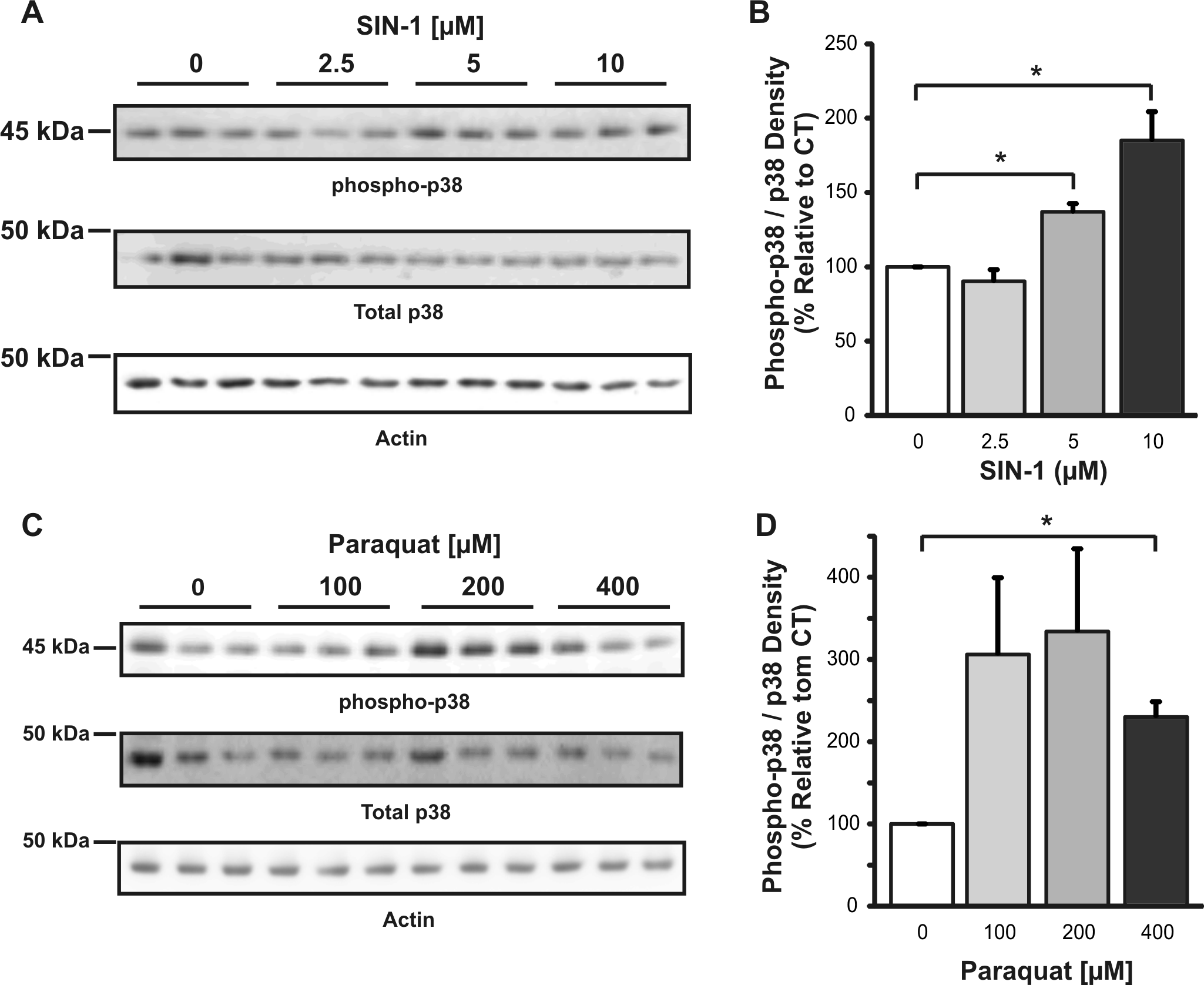

Supplement: Figure S3 — Immunoblot of phosphorylated p38 and total p38 in cortical cells treated with SIN-1 and paraquat. Western blot was carried out with 20 µg of cell lysates. Blots for SIN-1 and paraquat treatments are displayed in (A) and (C), respectively. These are representative of three separate experiments. The results indicate that the ratio of phospho-p38/total p38 was elevated in cells treated with 5 and 10 µM SIN-1 (B), supporting activation of p38 stress kinase. Treatment with 400 µM paraquat also caused a significant increase in phospho-p38/total p38 ratio (D). (TIF) [file pone.0061246.s003.tif]

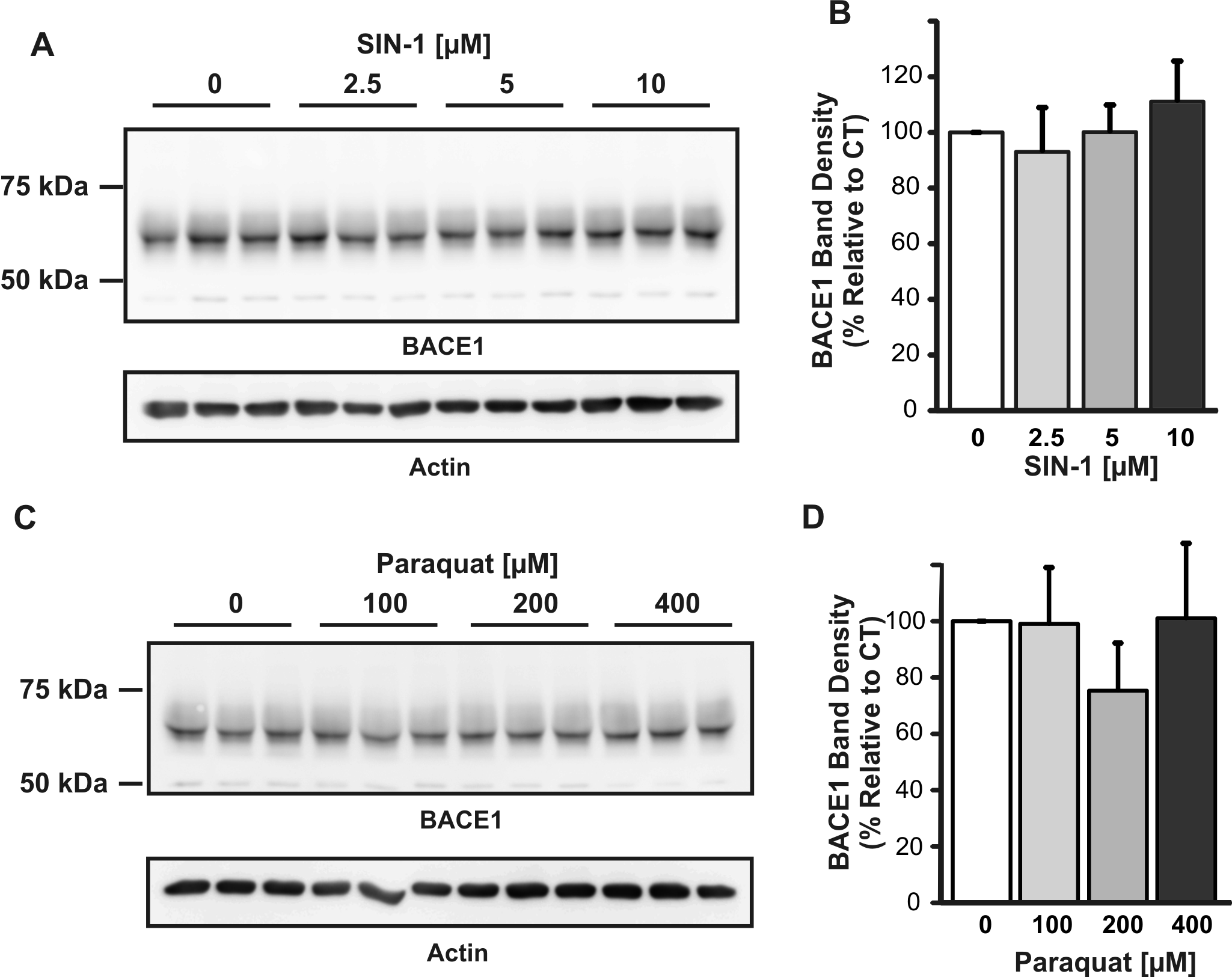

Supplement: Figure S4 — Analysis of BACE1 expression in primary cortical cells treated with SIN-1 and paraquat. Western blot was carried out with 20 µg of cell lysates using anti-BACE1 CT D10E5 antibody A. Representative immunoblot for SIN-1 treatment C. Representative immunoblot for paraquat treatment, respectively. Blots were re-probed for actin. B. and D. Densitometry analysis of blots for SIN-1 and paraquat treatments. The data represent averages of three individual experiments. BACE1 signal density was normalised to actin. No statistical difference was found between treated and control cells. (TIF) [file pone.0061246.s004.tif]

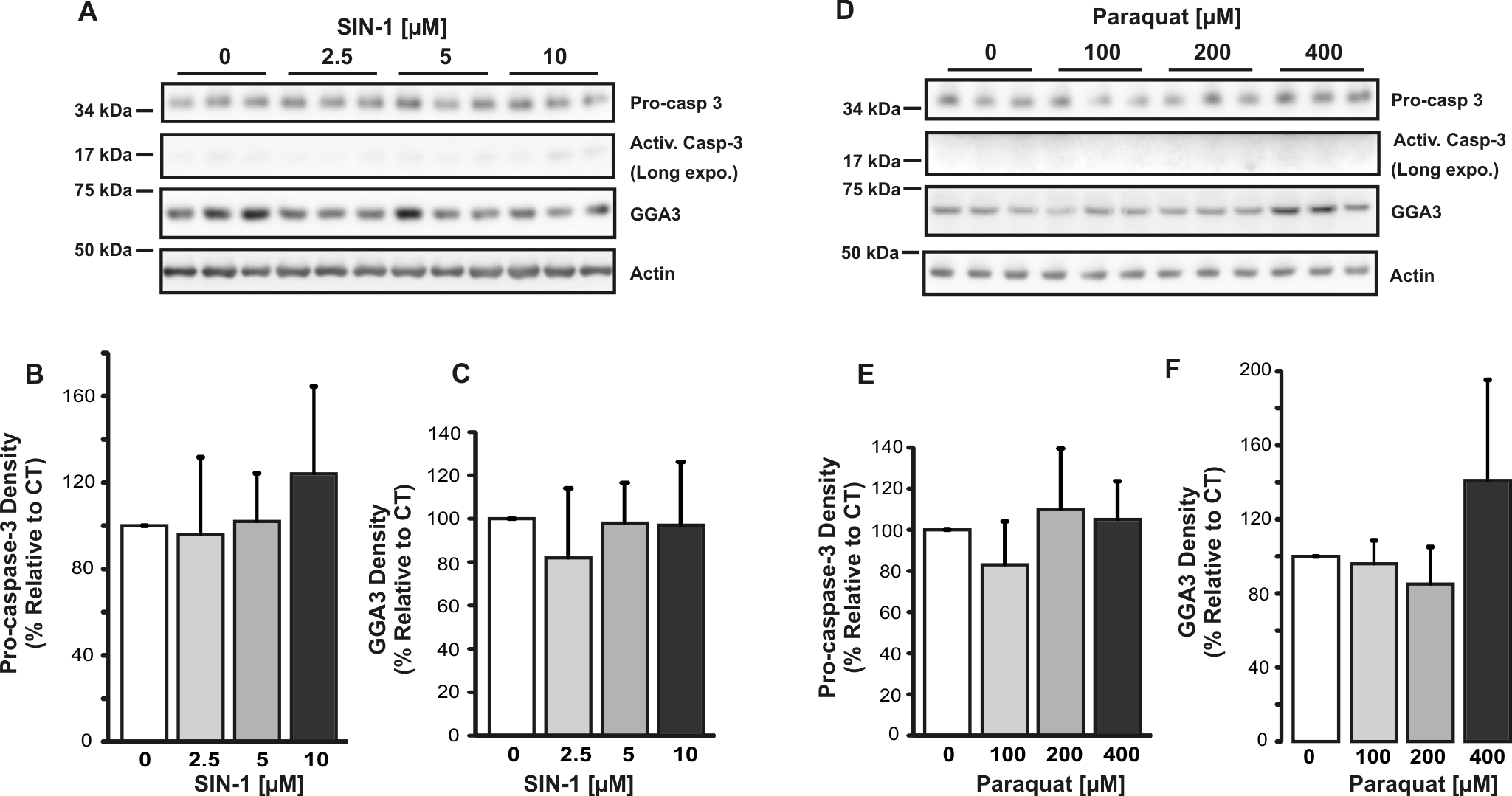

Supplement: Figure S5 — Immunoblot of caspase-3 and GGA3 in cortical cells treated with SIN-1 and paraquat. 20 µg of lysate was loaded per lane. A and D. Blots of caspase-3 and GGA3 for cells treated with SIN-1 and paraquat, respectively. B. Densitometry analysis of full-length pro-caspase-3 normalized to actin, for cells treated with SIN-1 (N = 3). C. Densitometry analysis of 75 kDa GGA3 signal normalized to actin, for cells treated with SIN-1 (N = 3). E. Densitometry analysis of full-length pro-caspase-3 normalized to actin, for cells treated with paraquat (N = 3). F. Densitometry analysis of 75 kDa GGA3 signal normalized to actin, for cells treated with paraquat (N = 3). There was significant change in pro-caspase 3 and GGA3 levels after either treatment with SIN-1 or paraquat at the indicated concentrations, suggesting that the cells were not undergoing apoptosis. (TIF) [file pone.0061246.s005.tif]

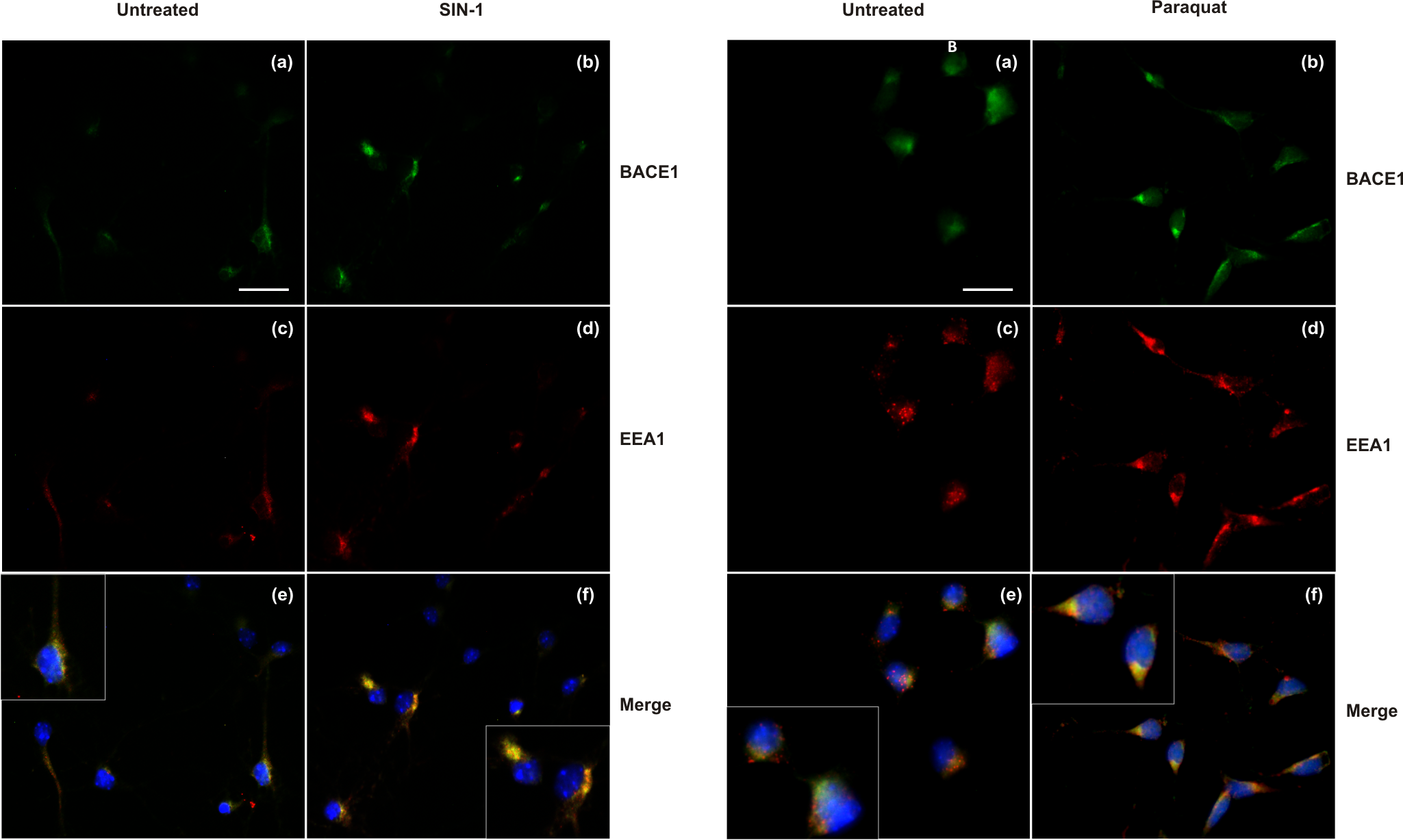

Supplement: Figure S6 — Immunofluorescence analysis of mouse primary cortical neurons treated with SIN-1 and paraquat. The left panel shows cells treated with SIN-1 and control. The right panel shows cells treated with paraquat and control. (a) and (b) show immunolabelling of BACE1 as punctate perinuclear staining (detected with mouse monoclonal antibody 61-3E7). (c) and (d) show detection of early endosomal marker EEA1. (e) and (f) are the merged images of BACE1 and EEA1. Partial colocalisation of BACE1 and EEA1 was observed in untreated cells (e), and this was increased in the cells treated with SIN-1 (left panel, f) or with paraquat (right panel, f). Bar = 20 µm. (TIF) [file pone.0061246.s006.tif]
