# Supplementary material for: Mild Oxidative Stress Induces Redistribution of BACE1 in Non-Apoptotic Conditions and Promotes the Amyloidogenic Processing of Alzheimer’s Disease Amyloid Precursor Protein
Source: PLoS One. 2013 Apr 17;8(4):e61246. doi: 10.1371/journal.pone.0061246 (PMC3629182; doi:10.1371/journal.pone.0061246)
Supplement: Methods S1 — (DOCX) [file pone.0061246.s007.docx]

**Supporting Information Methods**

*Cell Treatment*

Mouse primary cortical cells were plated at a density of 8 x 10^5^ cells per mL in 12-well poly-D-Lysine-coated tissue culture plate, and cultured for 6 days before treatment. Cells were treated with paraquat (Sigma) at 100, 200 or 400 μM for 6h; or SIN-1 (Sigma) for 24h, at a final concentration of 2.5, 5.0, or 10 μM. All compounds were prepared in culture medium supplemented with B27 Minus Antioxidants (Invitrogen). Control untreated cells were also cultured in culture medium supplemented with B27 Minus Antioxidants.

*Lactate Dehydrogenase Assay*

As paraquat compromises the function of mitochondria, the lactate dehydrogenase (LDH) assay was used as an alternative method to assess cell viability. This assay measures the release of LDH into the conditioned medium as cells lose membrane integrity. After treatment, 150 μL of conditioned medium was collected from each cell culture well and transferred to a 1.5 mL microfuge tube and centrifuged at 16,000 x g for 3 min. Then 100 μL of the supernatant was transferred to a 96-well plate; as a negative control, 100 μL of unconditioned medium served as a blank. The LDH reaction assay mix (Roche) was prepared according to the manufacturer’s instructions, and 100 μL added to each well. Absorbance was measured at 490 nm. An increase in absorbance compared to control reflects increased LDH activity, thus indicates increased cell death.
